# The Impact of Cortical Lesions on Thalamo-Cortical Network Dynamics after Acute Ischaemic Stroke: A Combined Experimental and Theoretical Study

**DOI:** 10.1371/journal.pcbi.1005048

**Published:** 2016-08-10

**Authors:** Joeri B. G. van Wijngaarden, Riccardo Zucca, Simon Finnigan, Paul F. M. J. Verschure

**Affiliations:** 1 Laboratory of Synthetic Perceptive, Emotive and Cognitive Systems (SPECS), Center for Autonomous Systems and Neuro-Robotics (N-RAS), Universitat Pompeu Fabra, Barcelona, Spain; 2 UQ Centre for Clinical Research, The University of Queensland, Royal Brisbane and Women's Hospital, Brisbane, Australia; 3 Institució Catalana de Recerca i Estudis Avançats (ICREA), Barcelona, Spain; Oxford University, UNITED KINGDOM

## Abstract

The neocortex and thalamus provide a core substrate for perception, cognition, and action, and are interconnected through different direct and indirect pathways that maintain specific dynamics associated with functional states including wakefulness and sleep. It has been shown that a lack of excitation, or enhanced subcortical inhibition, can disrupt this system and drive thalamic nuclei into an attractor state of low-frequency bursting and further entrainment of thalamo-cortical circuits, also called thalamo-cortical dysrhythmia (TCD). The question remains however whether similar TCD-like phenomena can arise with a cortical origin. For instance, in stroke, a cortical lesion could disrupt thalamo-cortical interactions through an attenuation of the excitatory drive onto the thalamus, creating an imbalance between excitation and inhibition that can lead to a state of TCD. Here we tested this hypothesis by comparing the resting-state EEG recordings of acute ischaemic stroke patients (N = 21) with those of healthy, age-matched control-subjects (N = 17). We observed that these patients displayed the hallmarks of TCD: a characteristic downward shift of dominant α-peaks in the EEG power spectra, together with increased power over the lower frequencies (δ and θ-range). Contrary to general observations in TCD, the patients also displayed a broad reduction in β-band activity. In order to explain the genesis of this stroke-induced TCD, we developed a biologically constrained model of a general thalamo-cortical module, allowing us to identify the specific cellular and network mechanisms involved. Our model showed that a lesion in the cortical component leads to sustained cell membrane hyperpolarization in the corresponding thalamic relay neurons, that in turn leads to the de-inactivation of voltage-gated T-type Ca^2+^-channels, switching neurons from tonic spiking to a pathological bursting regime. This thalamic bursting synchronises activity on a population level through divergent intrathalamic circuits, and entrains thalamo-cortical pathways by means of propagating low-frequency oscillations beyond the restricted region of the lesion. Hence, pathological stroke-induced thalamo-cortical dynamics can be the source of diaschisis, and account for the dissociation between lesion location and non-specific symptoms of stroke such as neuropathic pain and hemispatial neglect.

## Author Summary

The thalamus is involved in the relay and processing of most sensory information, and provides an interface between subcortical structures and the neocortex. However, disruptions in the subcortical communication with the thalamus are known to lead to thalamo-cortical dysrhythmia (TCD), which is linked to symptoms in a range of illnesses including Parkinson’s disease, neurogenic pain syndrome and tinnitus. Thus far, TCD has solely been interpreted in terms of changes within subcortical pathways, but here we investigate how cortical disturbances (*i*.*e*., ischaemic stroke) may affect thalamic function in a similar manner. We do so by analysing the electroencephalogram (EEG) of stroke patients with a cortical lesion, and show that their EEG power spectra display the characteristic features of TCD. We subsequently built a detailed spiking model of thalamo-cortical circuits to identify the local cellular, circuit, and network properties and dynamics that lead to the development of this stroke-induced TCD. Together, our results shed light on less-understood symptoms of stroke such as neuropathic pain and hemispatial neglect, help inform future brain monitoring and diagnostics post-stroke, and suggest potential new treatments for stroke and related neurological conditions.

## Introduction

The brain is a complex network of segregated, functionally specialised, yet densely interconnected regions that exchange and integrate signals with high spatio-temporal precision [1,2]. Pathological perturbations of a focal area within this network can spread to distant regions, and affect their function via maladaptive processes like diaschisis, that leads to dysfunction in a (otherwise seemingly healthy) brain region due to its connectivity to a distant, damaged brain region [3,4]. One of the most common focal perturbations of neuronal tissue are regionally localised lesions due to stroke. With ischaemic stroke (IS), blood supply to the brain is blocked by the occlusion of one or more cerebral arteries, resulting in a cascade of pathophysiological events that eventually lead to neuronal cell death [5]. In the early minutes to hours after an ischaemic episode, most clinical symptoms result from functional impairment within the infarcted (dying) “core” region, and the surrounding “penumbra”, an area also affected by ischaemia but potentially salvageable if blood flow is restored in a timely manner. In case penumbral regions are reperfused (*e*.*g*., by treatments delivered within hours of stroke onset), the symptomatology becomes more stable and specific, reflective of the loss of function within infarcted brain tissue [6,7]. Such symptoms can include sensory-motor deficits (*i*.*e*., sudden unilateral numbness or weakness in face or arm muscles), language disorders (*i*.*e*., aphasia), and/or cognitive deficits [8]. However, patients often suffer additional indirect and non-specific symptoms such as post-stroke pain and fatigue, hemispatial neglect, and mood-related disorders, that show an apparent dissociation with the lesion location, each with its own particular temporal dynamics in on- and off-set [9–12]. The mechanistic origins of many of these indirect symptoms of stroke are not well understood, and interventions remain undefined. Here we test whether stroke-induced pathological changes to the thalamo-cortical system (TCS) could serve as an underlying mechanism for generating such symptoms following an ischaemic episode.

The TCS displays a basic recurrent structure, reciprocally connecting thalamic nuclei with neocortical areas [13–15]. Disruptions within these circuits have been linked to symptoms in several neurological illnesses including Parkinson's disease, neurogenic pain syndrome, major depressive disorder, and tinnitus [16–19]. Associated symptoms are believed to develop as the result of an excessive expression of low-frequency bursting in thalamic nuclei and their propagation through the neocortex. This alteration of thalamo-cortical interactions is called thalamo-cortical dysrhythmia (TCD) [20]. TCD is caused by an imbalance of glutamatergic, cholinergic and/or GABAergic inputs to thalamic nuclei, creating hyperpolarised conditions that de-inactivate voltage-gated T-type Ca^2+^-channels, driving thalamic neurons into a low-frequency bursting mode of low-threshold calcium spikes (LTS) [21,22]. Synchronised bursting on a population level produces abnormal pathological oscillations in the θ-range that propagate through TC efferents, influencing their afferent regions in the neocortex. This low-frequency rhythmic activation of cortical areas creates local asymmetry of lateral cortical inhibition, also known as the “edge-effect”, which follows the characteristic shape of a Ricker wavelet, eliciting hyperactivity in neighbouring cortical modules [20,23]. Such hyperactivity, through increased coherence and cross-frequency coupling in the θ and β-range, has been linked to symptoms including Parkinsonian tremors and sensations of pain [24,25]. This link is further supported by the use of interventions that target thalamic nuclei and their afferent structures, such as deep brain stimulation (DBS), where repolarisation of pathological thalamic neurons can reinstate normal neurophysiological function [26]. Alternatively, surgical removal of the affected thalamic nucleus (central lateral thalamotomy) is associated with 70–95% pain relief for patients with neurogenic pain syndrome [25,27].

Thus far, TCD has solely been interpreted in terms of changes to the subcortical drive onto thalamic nuclei. Our hypothesis is that in ischaemic stroke, cortico-thalamic network dynamics are disturbed; in particular, the neocortical drive onto the thalamus is attenuated, leading to the emergence of TCD. We test this hypothesis by comparing the resting-state electroencephalograms (EEG) of ischaemic stroke patients (N = 21) with those of healthy age-matched controls (N = 17). We show that the EEG of stroke patients displays the characteristic features of TCD relative to controls, and we explain the genesis of this phenomenon with a computational model of the TCS. Using our spiking model, we demonstrate that lesions in cortical circuits resulting from stroke lead to excessive hyperpolarisation of thalamic neurons, switching them to a LTS bursting regime by de-inactivating I_T_-currents. This switch induces TCD dynamics and entrains thalamo-cortical pathways, propagating low-frequency oscillations into the neocortex, and through the edge-effect, could serve as a possible mechanism underlying the diverse symptomatology found after ischaemic stroke.

## Results

### EEG results

**Fig 1:**
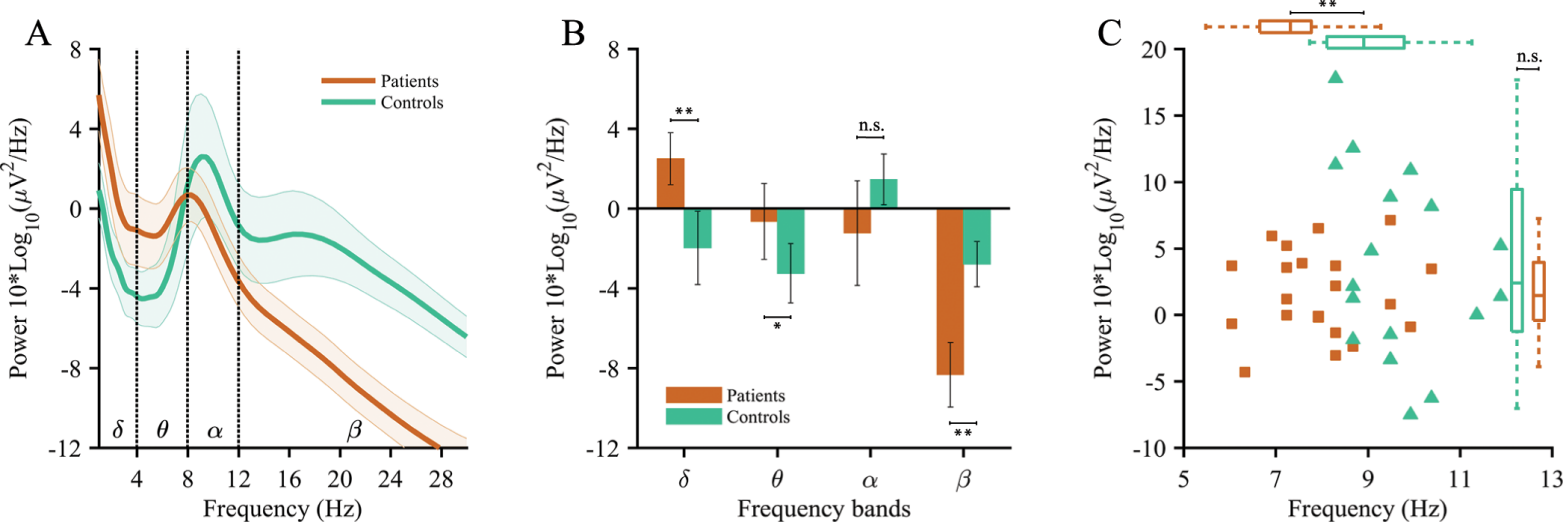
EEG power spectra. Wavelet convolution power spectra of the resting-state EEG data averaged over time and electrodes. (A) Mean spectra for patients (red) and controls (green), with the shaded area representing the 95%-confidence intervals. (B) Mean spectral energy (MSE) values (±s.e.m.) per frequency band, with significant differences in the δ (1–4 Hz), θ (4–8 Hz), and β (12–30 Hz) band. (C) Locations of the individual dominant α-peaks, showing a significant slowing into a lower frequency for patients (7.9±0.3 Hz) as compared to controls (9.7±0.3 Hz). Statistically significant differences marked as **p < .01, *p < .05, Wilcoxon rank-sum test.

Power spectra idiosyncratic to TCD display a slowing and occasional amplification of the dominant α-peak, increased power in the range of 1 to 30 Hz, and increased thalamo-cortical coherence [20,28]. Our stroke patients' EEG power spectra were consistent with this signature of TCD (Fig 1A). They displayed a significant increase in spectral power over the lower frequencies (Fig 1B) within the δ-band (*p* = .005) and θ-band (*p* = .027) compared to healthy matched controls. Within the higher frequency ranges, we observed no significant increases in the α-band (*p* = .078), while β-band power was significantly attenuated (*p* < .001). This latter finding is opposite from the EEG characteristics generally reported for TCD, suggesting a pathology-specific modulation of TCD in the β-band. The dominant α-peaks also shifted significantly (*p* < .001) towards a lower frequency for patients (7.9±0.51 Hz, range 6.1–10.4 Hz) compared to controls (9.7±0.56 Hz, range 8.3–11.9 Hz), whereas these peaks did not significantly differ (*p* = .378) in power (Fig 1C).

**Fig 2:**
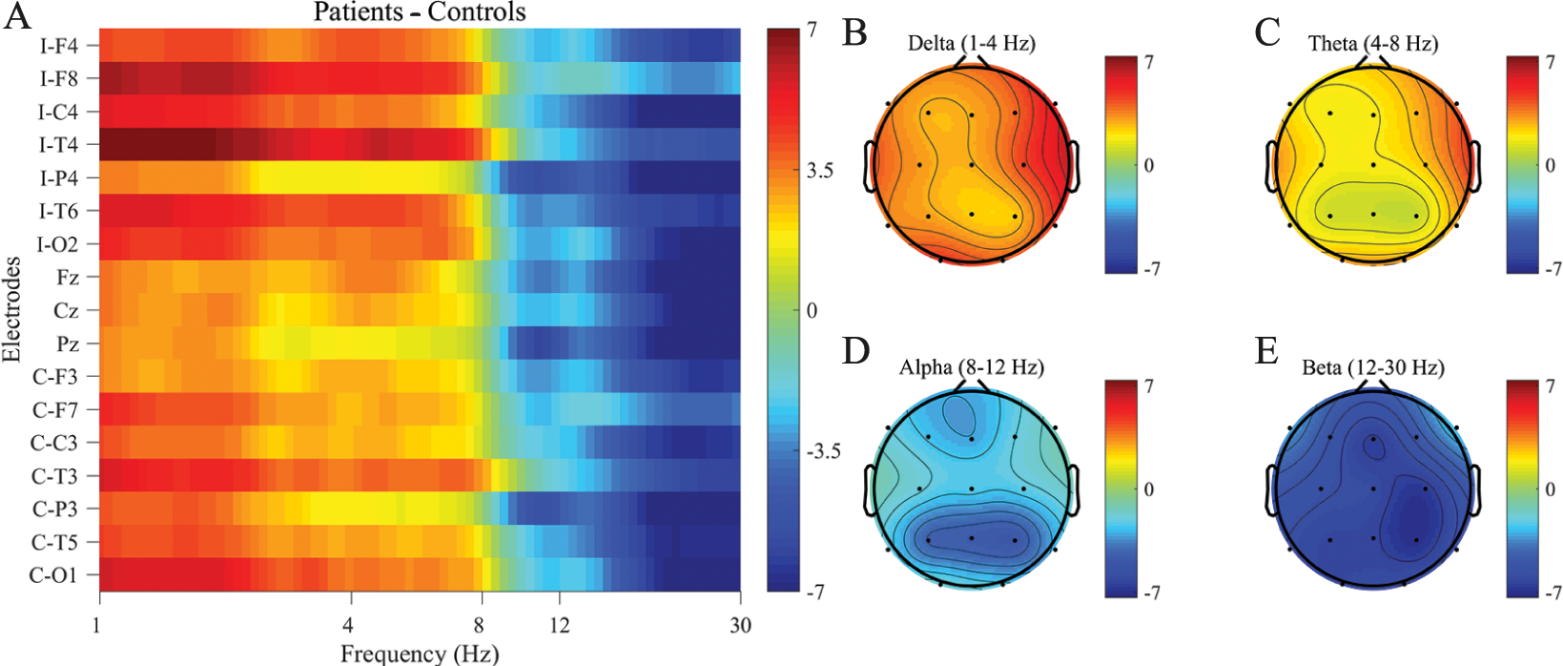
Topographical distribution of average differences between patients minus controls. Patients suffered a lesion in either of the two hemispheres, and in order to prevent averaging out hemispheric differences, all non-midline electrodes were swapped laterally for patients with a left-hemispheric lesion. Thus, the right hemisphere in the diagram should be considered ipsi-lesional while the left hemisphere is contra-lesional. (A) Average spectral power differences between patients minus controls, organised per electrode and marked with an I for ipsi-lesional and C for contra-lesional, with clear boundaries separating the different bands around 8 and 12 Hz. (B-E) Topographical distribution of binned power differences for the four separate frequency bands: δ, θ, α and β respectively. Differences in the δ and θ-band are qualitatively similar, where patients display an increase over the lateral ipsi-lesional electrodes (F6, C4, T4 and T6) together with the contra-lesional electrode T3 and occipital electrodes O1 and O2. There are no differences in the α-band, and the decrease of β-band activity is spread across all scalp electrodes.

The analyses thus far spanned the average over all electrodes, but by arranging the spectral power differences over all frequencies against electrode positions, the topological distribution of differences between stroke patients and control subjects becomes evident (Fig 2A). This distribution in mean spectral energy (MSE) for the four different bands revealed localised increases in the δ and θ-band, predominantly at the lateral ipsi-lesional electrodes (F8, C4, T4 and T6), and to a lesser extent contra-lesional (T3), together with the occipital electrodes (O1 and O2) (Fig 2B and 2C). The significant decrease of β-band power for patients was not localised, but was instead distributed across all scalp electrodes, while there were no differences between patients and controls in the α-band (Fig 2D and 2E). Individual patient comparisons with the control group average can be found in the SI (S1 Fig).

### Modelling thalamo-cortical dynamics post-stroke

**Fig 3:**
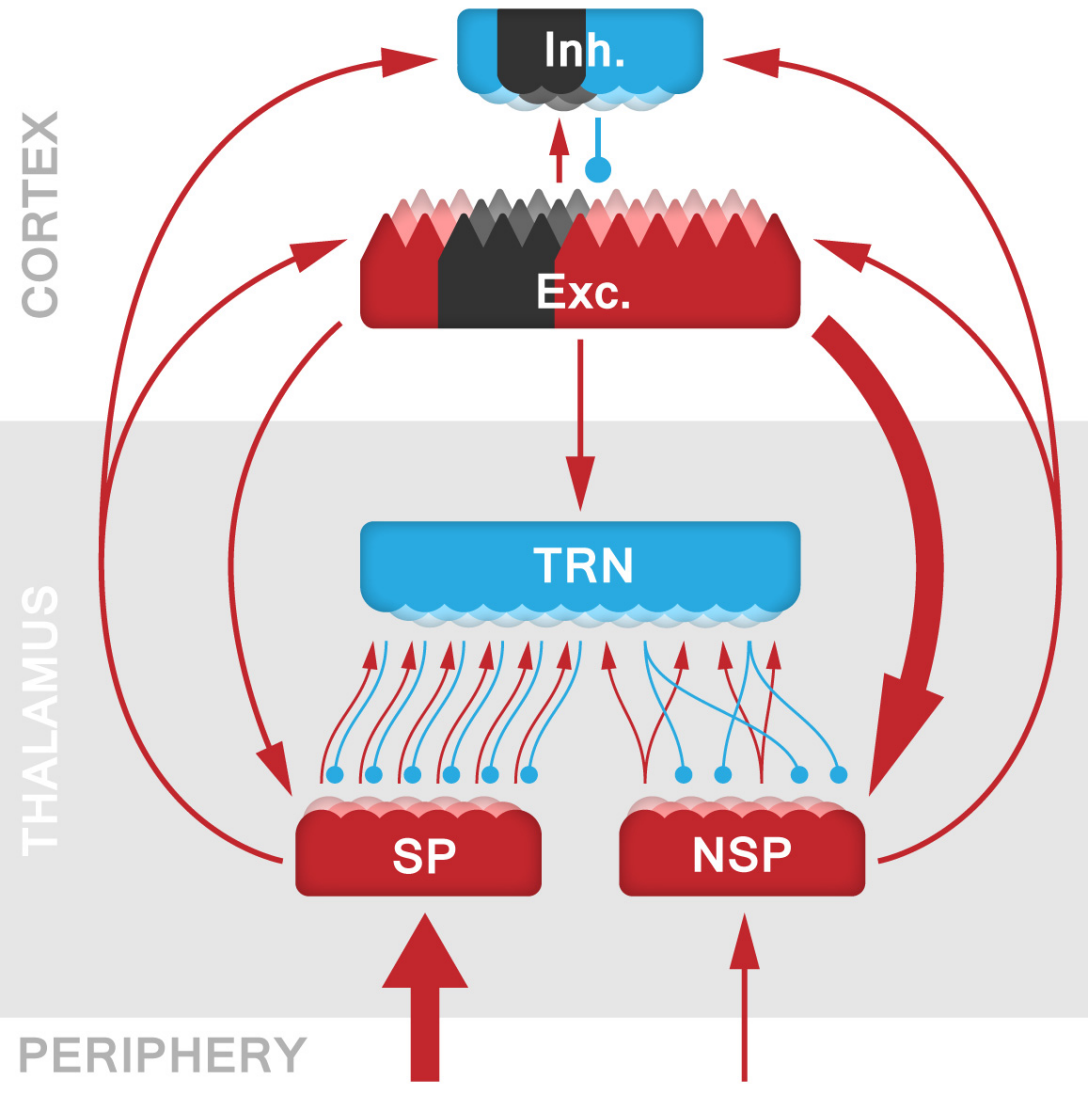
Schematic representation of the TCS model architecture. The thalamic component of the model comprises specific (SP; corresponding to the ventral posterior complex of the thalamus in the somato-sensory domain), non-specific (NSP; comparable to the intralaminar nuclei) and the thalamic reticular nucleus (TRN). The cortical layer includes both excitatory (Exc.) pyramidal cells and inhibitory (Inh.) interneurons. Excitatory connections are marked with red lines terminating in arrowheads, while inhibitory connections are blue with circles indicating their synaptic terminals. The simulated stroke is implemented by removing the cortico-cortical connections from a specific cortical area, marked in black. All the model’s parameter values can be found in Tables 1–3.

In order to identify the mechanistic origins of stroke-induced TCD, we built a spiking model of the TCS, based on our previous work [29], and calibrated with the EEG data (see methods for further details). Our model captures the reciprocal connectivity between a cortical area (CRX), a modality-specific thalamic relay nucleus (SP), a multi-modal and non-specific thalamic nucleus (NSP), and the inhibitory thalamic reticular nucleus (TRN) (Fig 3). With this model we wanted to: (*i*) show that cortical lesions produce TCD-like dynamics post-ictal within the thalamus, as observed after peripheral deafferentation in modelling neurogenic pain [29]; (*ii*) explain the downward shift of dominant α-peaks in the cortical power spectra; and (*iii*) explain the stroke-specific decrease of spectral power within the β-band.

**Table 1:** Network connectivity values, based on [29].

Connection	Type	g_s_ (mS)	Connectivity	τ_s_	Delay (ms)
Input-SP *→* SP	Exc.	0.005	1-to-1	10	0
Input-NSP *→* NSP	Exc.	0.005	1-to-1	10	0
SP *→* TRN	Exc.	0.018	1-to-1	10	3
TRN *→* SP	Inh.	0.35	1-to-1	75	3
TRN *→* NSP	Inh.	0.18	1-to-many (0.15)	75	3
NSP *→* TRN	Exc.	0.015	1-to-many (0.15)	10	3
E *→* TRN	Exc.	0.02	8-to-1	7	7
E *→* SP	Exc.	0.007	8-to-1	7	7
E *→* NSP	Exc.	0.02	8-to-1	7	7
SP *→* E	Exc.	0.002	1-to-8	7	7
NSP *→* E	Exc.	0.8	1-to-many (0.03)	7	7
E *→* E/I	Exc.	0.3	all-to-all	-	-
I *→* I/E	Inh.	1	all-to-all	-	-

**Table 2:** Thalamic neuron parameter values.

	SP	NSP	TRN
Number (#)	100	100	100
k_E_	1	1	1
k_I_	0.1	0.1	0.1
V_0_ (mV)	-35	-35	-35
V_reset_ (mV)	-50	-50	-50
V_L_ (mV)	-65	-65	-65
V_E_ (mV)	0	0	0
V_I_ (mV)	-85	-85	-85
V_T_ (mV)	-66	-66	-64
C (μF/cm^2^)	2	2	2
g_L_ (mS/cm^2^)	0.035	0.035	0.035
g_T_ (mS/cm^2^)	0.07	0.07	0.07
$\tau_{h}^{-}$ (ms)	20	20	40
$\tau_{h}^{+}$ (ms)	100	100	100

**Table 3:** Cortical neuron parameter values.

	Excitatory	Inhibitory
Number (#)	800	200
a (ms)	0.02	0.02 + 0.08r
b (ms)	0.2	0.25–0.05r
c (mV)	-65 + 15r^2^	-65
d (mV/ms)	8–6r^2^	2

r; random variable, uniformly distributed between 0–1 for each neuron

**Fig 4:**
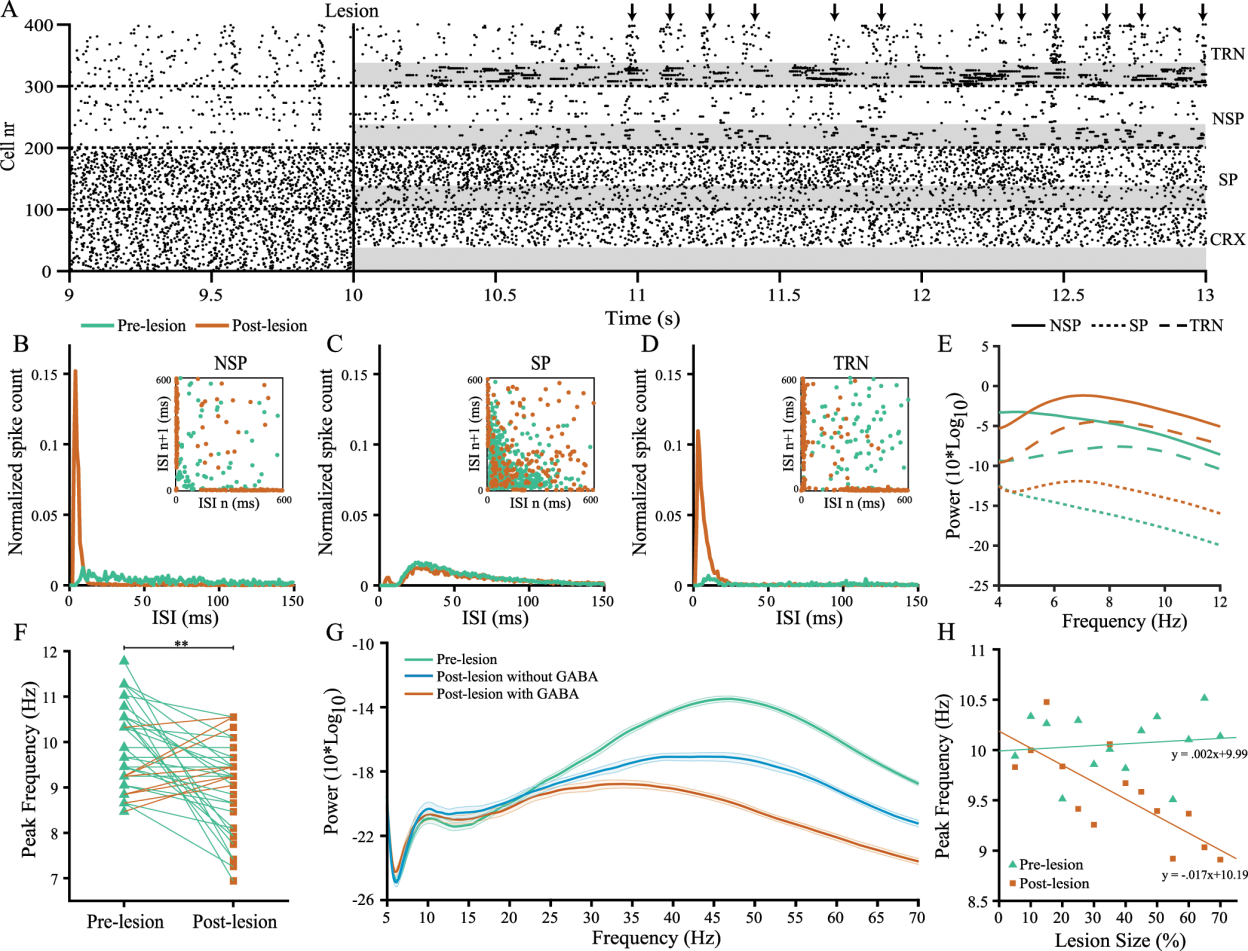
Model results. (A) Rasterplot of spikes for a single run of the model. From top to bottom: 100 neurons of the three thalamic nuclei TRN, NSP, SP plus the excitatory cortical population (CRX). A lesion was introduced after 10 seconds of real-time simulation time by deafferentating 30% of the cortical neurons, resulting in a loss of driving inputs for the thalamic neurons marked in grey. Arrows indicate entrained oscillations across the different nuclei. (B-D) Inter-spike intervals (ISI) for the NSP, SP and TRN, respectively. Most thalamic neurons pre-lesion are in a tonic-firing mode, with evenly distributed ISIs. After the lesion, the NSP and TRN switch to a bursting regime, with the majority of neurons having an ISI below 20 ms indicating intra-burst spikes, followed by longer inter-burst intervals. (E) Power spectra for the NSP (solid line), SP (dotted line) and TRN (dashed line) in the lower frequency range. All thalamic nuclei display a substantial increase of spectral power in the low-frequency range, peaking between 6 and 8 Hz, with the strongest oscillations present in the NSP and TRN. (F) Peak frequencies of the cortical power spectra in the range of 6–13 Hz, with an overall downward shift into a lower frequency post-lesion (9.2±0.38 Hz) compared to pre-lesion (9.9±0.44 Hz). Line colours indicate the highest corresponding frequency. (G) Average power spectra of the cortical component with and without the extrasynaptic increase of GABA post-lesion, with shaded areas representing the 95%-confidence intervals. The lesion affects γ-band power in both models post-lesion, but with an extrasynaptic increase of GABA, β-band power is further suppressed from 23 Hz onwards, comparable to the decrease for patients in their EEG. (H) Peak frequencies for different lesion sizes, averaged over 10 simulations per step. As the lesion size increases, the dominant α-peaks slow down towards a lower frequency post-lesion. Statistically significant differences marked as **p < .01, Wilcoxon-rank sum test.

During a state of wakefulness, most thalamic neurons function under relatively depolarised conditions and, with inactive *I*_*T*_-currents, respond to excitatory input with sustained firing of unitary spikes [30]. These dynamics are captured in the model pre-lesion, with most neurons in a tonic firing mode and little to no coherence across the nuclei (Fig 4A–4D). We then approximated the conditions of stroke by lesioning thirty percent of the cortical population within the model, partially removing driving inputs to the thalamic nuclei. We observed that these conditions result in hyperpolarisation relative to baseline, where T-type Ca^2+^-channels are de-inactivated and neuronal discharges become LTS bursts rather than single spikes (Fig 4B–4D). As the recurrent cortical projections predominantly terminate onto the non-specific and reticular nuclei, bursting is initially confined to a subcircuit within the NSP and TRN. However, bidirectional divergent connectivity between these nuclei promotes the propagation of bursting activity to non-affected regions. Once a critical mass of neurons enter a bursting regime, dynamic recruitment of the TRN leads to hyperpolarised conditions for an increasing number of neurons in the NSP. This alternating interplay between the NSP and TRN synchronises neural activity across the different nuclei in the θ-range (Fig 4A). The SP however is driven largely by peripheral sensory inputs, and a cortical lesion attenuates excitatory input for this nucleus to a much lesser extent. These neurons remain functional under relatively depolarised conditions in a tonic firing mode, and only a minor subset of neurons display bursting behaviour (Fig 4D).

Synchronised bursting is characterised on a population level by a substantial increase in low-frequency oscillations post-lesion for all thalamic nuclei, peaking between 6 and 8 Hz, most prevalent in the NSP and TRN (Fig 4E). With the propagation of such oscillations through TC efferents, dominant α-peaks of the cortical power spectrum are driven from 9.9±0.44 Hz towards a lower frequency of 9.2±0.38 Hz post-lesion (*p* = .002) (Fig 4F), coherent with slowing of the α-peaks found in the EEG spectra of patients. Parametrisation of the lesion size parameter shows its linear relationship with this shift, where an increased number of lesioned neurons correspond with a greater slowing of dominant peaks (Fig 4H).

During an ischaemic episode, the brain's response is to increase extrasynaptic levels of GABA as a neuroprotective mechanism, inhibiting all glutamate mediated neuronal activity in order to suppress rising levels of excitotoxicity, and prevent additional tissue damage [31]. To capture this extrasynaptic increase of GABA, a constant inhibitory current was applied to all cortical neurons post-lesion following a slow onset. The cortical lesion in the model resulted in a decrease of γ-band activity, but adding this inhibitory current further suppressed γ-band power (Fig 4G). Moreover, high-β-band activity also decreased, creating divergence between the pre- and post-lesion spectra from 23 Hz and upwards (Fig 4G).

## Discussion

We have addressed the question whether thalamo-cortical dysrhythmia develops after ischaemic stroke. We hypothesised that cortical lesions, for instance due to ischaemic stroke, lead to an attenuation of the neocortical drive onto the thalamus, changing thalamic cellular and circuit dynamics such that thalamo-cortical dysrhythmia (TCD) emerges. Our analysis of the EEG of stroke patients confirms our hypothesis and shows the characteristic TCD shifts in the power spectrum. The electrophysiological data display high similarity with the well-established EEG characteristics found in the wide range of disorders previously linked with TCD [23,25,28].

The most prominent overlap is the slowing of the dominant α-peak, together with an increase in spectral power over the lower frequencies, both in the δ-band and θ-band. These results are consistent with changes in the EEG power spectra generally observed in stroke. Rapid appearance of high-amplitude oscillations in the δ-band is highly typical of ischaemic stroke, and is often preceded by both a slowing of the α-peaks and attenuation of β-band oscillations in cases of intermediate to severe ischemia (see [32] for a review). This β-band attenuation, also observed in our patients, is opposite from what is generally reported for TCD, and could be related to increased levels of extrasynaptic GABA during the acute phase of stroke [31,33]. Indeed, approximating this increase in our computational model not only further suppressed γ-band power, but also decreased high-β-band power from 23 Hz upwards, comparable to what we observed in the stroke patients.

Our patients' EEG were acquired during the acute phase, but are consistent with Dubovik *et al*. [34], who previously reported a similar downward shift in α-peaks, and comparable modulations in all frequency bands in EEG recorded 3 months post-stroke. The authors emphasise changes in the spatial distribution of α-band power, their coherence, and relationship to the symptomatology, supporting the notion of diaschisis following stroke [4]. Here we determine more precisely the similarities between such stroke-induced diaschisis and thalamo-cortical dysrhythmia, identifying a possible substrate underlying the dynamic changes to the TCS after stroke.

We subsequently modelled the dynamics of the TCS by which cortical insults lead to the genesis of pathological thalamic low-frequency oscillations and TCD-like phenomena. Using a spiking model of the thalamo-cortical system, we managed to identify core candidate mechanisms responsible for the attenuation found in the power spectra. Indeed, with a lesion in the cortex, excitatory inputs to thalamic nuclei are deafferentated, resulting in disfacilitation and cell membrane hyperpolarisation that in turn leads to the de-inactivation of T-type Ca^2+^-channels and a switch to LTS bursting. While initially confined to a subcircuit, activity spreads through divergent connectivity in the intrathalamic pathways, enforcing synchronised oscillations on a population level in the high-θ range. The influx of such slow oscillations through thalamic efferents into the cortex drives the dominant α-peaks into a lower frequency. We predict that this entrainment of thalamo-cortical circuits will lead to further effects such as those observed in TCD.

While the propagation of low-frequency thalamic oscillations into the neocortex is common in a state of drowsiness or sleep, they have pathological properties in an awake state through an ‘’edge-effect‘’ [20,23]. Oscillations influence local interneurons and create a ring of imbalance in cortico-cortical inhibition surrounding the target area, resulting in waves of hyperactivity in neighbouring regions. During the acute phase of stroke, this mechanism could be involved in positive post-stroke symptoms that have a relatively short onset, such as post-stroke pain or fatigue, when similar pathways involved in neurogenic pain are entrained [23,25]. In contrast, TCD could additionally be associated with rapid emerging negative symptoms. For example, given the important role of the TRN in visual information processing and attention, a strong increase of reticular inhibition within the visual thalamo-cortical circuit could disrupt top-down inhibitory networks between prefrontal areas and thalamus, resulting in negative symptoms such as hemispatial neglect [11,35]. On a network level, prolonged pathological hyperactivity could affect the dynamics of cortical circuits during the chronic phase. While less understood, plasticity could lead to a dynamic reorganisation of cortical networks, resulting in chronic symptoms with a much longer onset, like depression, that typically develop after several months [18,36].

The approach to TCD has thus far been to examine subcortical drives onto the thalamus, either sensory input coming from peripheral nerve fibres (*e*.*g*., with neurogenic pain), or inhibitory projections from the basal ganglia (*e*.*g*., with Parkinson's disease) in the pallido-thalamic tract [37,38]. Our results suggest that TCD can extend into a much broader range of disorders, where any form of cortical tissue damage could lead to the development of TCD-like dynamics within the TCS. Cortical trauma can disrupt cortical function over spatially distributed areas through diaschisis, altering the physiological state of distant regions, and modulate their activity [34]. This process not only affects cortico-cortical connections, but also has a direct impact on thalamic function through cortico-thalamic pathways. While this change initially affects thalamic function locally, once it reaches the dynamical attractor state of TCD, pathological oscillations will persistently propagate through circuits of the neocortex, affecting increasingly more distal areas. Consistent with this notion is a recent link between TCD and severe sepsis and septic shock [39]. Sepsis is a severe inflammatory response to an infection, leading to sepsis-associated encephalopathy due to brain-cell damage, impaired neurotransmission, and neurodegeneration [40–42]. As a result, patients often suffer from a rapid decline in cognitive function, including impaired memory, attention, and visual-spatial processing. It has been proposed that the neuropathological processes following sepsis additionally lead to the development of TCD, as the patients' EEG display the distinctive slowing of the dominant α-peak into high-θ range [39].

TCD is generally identified using either non-invasive EEG or MEG, a method that has been validated with intracranial recordings in patients undergoing stereotactic surgery [16,43,44]. While our current study does not include direct thalamic recordings, EEG allowed us to identify TCD post-stroke, but the exact relationship between TCD as a mechanism and the genesis of non-specific symptoms remains unclear, and thus requires further investigation. One direction is connecting extensive clinical assessment of stroke patients, covering cognitive domains such as neglect and attention, chronic pain, and mood-related impairments, with TCD-specific characteristic EEG features. Using high-density recordings, different source localisation techniques could furthermore be used to localise specific features such as α-peak shifts and low-frequency power modulations in relation to specific symptoms. Once this link between TCD and stroke-associated symptoms becomes more evident, it would open new avenues of interventions proven effective to treat TCD. This includes stereotactic thalamotomy or tractotomy, together with deep brain stimulation, used to treat Parkinson's disease and neurogenic pain syndrome [26,43,44].

### Conclusions

By combining the analysis of electrophysiological (scalp EEG) recordings from stroke patients with a computational model of thalamo-cortical circuitry, we showed that characteristic, post-stroke EEG abnormalities can be accounted for in terms of TCD. We also identified the substrate of diaschisis and thalamo-cortical dysrhythmia, where stroke not only affects cortical function locally, but furthermore perturbs distributed cortical and thalamo-cortical networks (and thus brain regions distant to the lesion), making it a prime target for the emerging field of network medicine [45,46]. By understanding the adverse impact of stroke on brain network function in terms of TCD, we advance an alternative perspective on the genesis of diaschisis and the non-specific symptomatology of stroke. Similar disruptions within the TCS are present in other thalamo-cortical disorders, suggesting a common underlying mechanism among different neuropathologies, which can produce different symptoms depending on the affected thalamo-cortical circuit(s).

## Methods

### Clinical data

Approval to carry out the study was obtained from the local University and Hospital Human Research Ethics Committees. Written informed consent from each patient or substitute decision-maker was obtained. Participants for this study included stroke patients (N = 21, 11 female; mean age 72 years, range 38–85), all suffering from acute middle cerebral artery (MCA) stroke and recruited from the Royal Brisbane and Women's Hospital in Brisbane, Australia. Stroke was assessed using acute computed tomography (CT) scans, followed by magnetic resonance imaging (MRI) in six cases. EEG data were acquired at the patient's bedside in the acute phase, approximately 69 hours (range 21–99) after stroke onset and used previously to optimise electrode placement in stroke prognostics [47]. In case patients woke up with stroke symptoms, time of stroke onset was defined as the midpoint between bedtime and time of waking up. Patient demographic information can be found in the SI (S1 Table). EEGs were recorded using a NicOne Brain Monitor (Natus Medical Inc.), recording at a sampling rate of 500 Hz and using 19 Ag/Ag-Cl electrodes (Nicolet; Natus Medical Inc.), placed according to the international 10–20 system. The reference data came from healthy age-matched controls (N = 17, 8 female; mean age 68 years, range 60–80) with no cognitive impairments or history of depression and/or anxiety, previously gathered for [48]. EEGs were recorded using an elasticised quick cap with 32 Ag/Ag-CL electrodes (Neuromedical supplies), sampling at 500 Hz and digitised by a Neuroscan Synamps amplifier.

### Topology model

A schematic of the model’s architecture is shown in Fig 3. Thalamo-cortical dynamics were simulated using the open-source IQR neural network simulator [49] over a total of 40 separate simulations, each with a random initialisation of thalamo-cortical and cortico-cortical connectivity. The temporal integration of each time step was 1 ms with a total recording time of 20 seconds real-time. The model’s network builds on [29] with similar thalamic circuitry, but we extended the Poissonian cortical input to a layer that includes both excitatory and inhibitory neurons and displays cortical dynamics (see neuron model below).

The thalamic component consists of three classes of nuclei: (*i*) a modality-specific relay nucleus (SP), corresponding to the ventral posterior complex of the thalamus in the somato-sensory domain; (*ii*) a non-specific higher-order nucleus (NSP), comparable to the intralaminar layer; and (*iii*) the inhibitory reticular (TRN) nucleus [30,50]. The excitatory SP and NSP nuclei are distinguished in terms of their distinct connectivity patterns within the model. The SP is characterised by parallel one-to-one connections (*e*.*g*., a single source neuron is connected to a single target neuron) with the TRN and is driven primarily by peripheral sensory inputs [51]. Conversely, the NSP receives most of its input from cortical layer VI and has strong divergent one-to-many connections (*e*.*g*., a single source neuron is connected to all target neurons with a given probability) with the TRN and cortex [52,53]. These anatomical properties are captured by the connectivity values between a source and target neurons of the model (Table 1). The random peripheral input for the SP and NSP consisted of Poisson spike trains with a given spiking probability *P* per iteration. We tuned the values of *P*_*SP*_ = 0.5 and *P*_*NSP*_ = 0.35 such that the nuclei at resting-state fire within a physiological range, while maintaining dominance of peripheral input for the SP [15].

### Neuron model

Characteristic membrane properties of thalamic neurons include polarisation-dependent inactivation of T-type Ca^2+^-channels, capable of producing low-threshold calcium spikes under hyperpolarised conditions. The de-inactivation of such calcium currents is modelled by adding a slow variable, *h*, to the classical conductance-based leaky integrate-and-fire dynamics, suggested by [54]. TC neurons were modelled as single-compartment cells, where changes in the membrane potential depend on an input current, *I*_*in*_, calcium currents, *I*_*T*_, and a constant conductance leak current *I*_*L*_:

$$C\frac{dV}{dt}=I_{in}-I_{L}-I_{T}$$ (1)

The calcium currents depend on the inactivation level, *h*, that relaxes to zero at depolarised levels when *V* > *V*_*h*_, or approaches unity under hyperpolarised conditions, given time constants *τ*_*h*_^*-*^ and *τ*_*h*_^*+*^:

$$I_{T}=g_{T}m_{\infty }h\left(V-V_{T}\right)$$ (2)

$$\frac{dh}{dt}=\{\begin{array}{c} -h/\tau_{h}^{-}\\ \left(1-h\right)/\tau_{h}^{+} \end{array}\begin{array}{c} \left(V>V_{h}\right)\\ \left(V<V_{h}\right) \end{array}$$ (3)

The input current, *I*_*in*_, is dependent on all excitatory minus inhibitory synaptic currents, g_E/I_, calculated as the sum over the multiplication of the connectivity matrix, *W*_*ij*_, with a dichotomous spiking vector, s_j_, that indicates spiking neurons (eqs 4 and 5). The result is weighted with a gain, *k*_*E/I*_, and follows exponential decay dynamics with time constants *τ*_*E/I*_. All parameter values can be found in Table 2 and were chosen based on [29,54].

$$I_{in}=g_{E}\left(V-V_{E}\right)-g_{I}\left(V-V_{I}\right)$$ (4)

$$\frac{dg_{E/I}}{dt}=\frac{-g_{E/I}}{\tau_{E/I}}+k_{E/I}\sum_{j=1}^{N}W_{ij}s_{j}$$ (5)

The cortical population includes 800 excitatory and 200 inhibitory neurons and were modelled as quadratic integrate-and-fire cells [55], according to a set of differential equations that capture the membrane potential, *v*, and a state variable, *u*:

$$\frac{dv}{dt}=0.04v^{2}+5v+140-u+I$$ (6)

$$\frac{du}{dt}=a\left(bv-u\right)$$ (7)

A cell is considered to spike when the membrane potential reaches a threshold of 30 mV, after which *v* ← *c* and *u* ← *u* + *d* capturing after-spike repolarisation. Parameters *a*, *b*, *c* and *d* were chosen to produce firing behaviour similar to regular spiking (RS) neurons (Table 3), in concordance with [55]. We explored the parameter space for the intrinsic input, *I*, to find the optimal combination of values (*I*_*E*_ = 6.7 and *I*_*I*_ = 2.7) that reproduces power spectra with dominant α-peaks comparable with those found in the healthy EEG data.

After simulating the thalamocortical system in a healthy state, a structural lesion was approximated by deafferentating 30% of adjacent excitatory and inhibitory cortical neurons, disconnecting all connections to and from these neurons. The impact of different lesion sizes on network behaviour can be found in Fig 4H. To retain comparability with the human EEG data, a noise constraint was applied by omitting simulations (4 out of 40) with dominant α-peaks pre-lesion outside boundaries given by the minimum and maximum peak frequencies of the control group's EEG.

### Data analysis

Signal processing and analyses were performed offline using MatLab (Mathworks, Natrick, Ma, USA) and the toolbox EEGLAB [56], together with custom in-house scripts. All data were bandpass filtered between .5 and 35 Hz (12dB/octave) and re-referenced to the common average of all electrodes. The first 45 epochs of 2048 ms were selected that included no clear artefacts or extreme values of ±75 μV. Only the following 17 electrodes common across both groups were included for further analyses: F3, F4, F7, F8, Fz, C3, C4, Cz, P3, P4, Pz, T3, T4, T5, T6, O1 and O2.

Wavelet convolution was used to decompose both the EEG and model time-series data into their underlying oscillatory components [57]. At first, a set of complex Morlet wavelets were computed, scaling logarithmically from 1 to 35 Hz for the EEG and from 1 to 80 Hz for the model, normalised by the wavelet's maximum value. With an equal amount of cycles, higher frequencies span a shorter time-window than lower frequencies, hence the amount of cycles used scaled in accordance with their frequency. Next, the fast Fourier transformation (FFT) of the signal was multiplied in complex space with the FFT of each wavelet, and taking the inverse FFT gives an analytical signal in the time-domain. Taking the square of the real part of this signal provides the amplitude at each time-frequency point. Input for the convolution were pre-processed time-series signals of the EEG, described in detail in [47], while a local field potential (LFP) was used for the model, calculated as the combined membrane potentials for all non-lesioned neurons per population. The result of the convolution was averaged over time and binned into four commonly used frequency bands: delta (δ; 1.0–4.0 Hz), theta (θ; 4.2–7.9 Hz), alpha (α; 8.3–11.9 Hz) and beta (β; 12.4–30.6 Hz), giving the mean spectral energy (MSE) per band.

### Statistical analysis

All statistical testing was done using the non-parametric Wilcoxon rank-sum test, with significant differences marked as **p < .01, *p < .05. Values reported in text are always means ±s.e.m. and shaded areas in figures represent the 95%-confidence intervals.

## Supporting Information

S1 Table Clinical description of patients. (DOCX)

S1 Fig Topographical distribution of individual differences in spectral power. A difference was computed between each patient individually minus the control group average in the combined frequency range of δ and θ (1–8 Hz). Stroke patients show high heterogeneity in affected regions. There are either unilateral or bilateral increases (*i*.*e*., patient 2 versus 3) that can be localized or spread across the scalp (*i*.*e*., patient 7 versus 12), affecting different cortical areas such as frontal regions (patient 14), visual areas (patient 2), motor areas (patient 20) or parietal regions (patient 12). Despite these individual differences, we do observe global changes across all patients, such as a shift in the α-peaks, suggesting the development of TCD.
(EPS)
