# Supplementary material for: The Impact of Cortical Lesions on Thalamo-Cortical Network Dynamics after Acute Ischaemic Stroke: A Combined Experimental and Theoretical Study
Source: PLoS Comput Biol. 2016 Aug 10;12(8):e1005048. doi: 10.1371/journal.pcbi.1005048 (PMC4979968; doi:10.1371/journal.pcbi.1005048)
Supplement: S1 Table — (DOCX) [file pcbi.1005048.s001.docx]

**Supporting Information**

**S1 Table. Clinical description of patients.**

| **Patient ID** | **Age** | **Gender** | **Time since stroke (h)** | **Affected hemisphere** | **Peak Freq.** |
| --- | --- | --- | --- | --- | --- |
| 1 | 56 | Male | 74 | Right MCA | 6.1 |
| 2* | 82 | Female | 75 | Left MCA | 7.9 |
| 3* | 84 | Female | 76 | Left MCA | 7.9 |
| 4* | 38 | Female | 57 | Left MCA | 9.5 |
| 5* | 84 | Female | 76 | Right MCA | 7.2 |
| 6 | 82 | Male | 68 | Left MCA | 6.3 |
| 7 | 45 | Male | 74 | Left MCA | 8.3 |
| 8 | 74 | Female | 68 | Right MCA | 7.9 |
| 9 | 66 | Female | 72 | Right MCA | 9.9 |
| 10 | 85 | Male | 73 | Right MCA | 6.1 |
| 11* | 74 | Female | 71 | Left MCA | 6.9 |
| 12 | 81 | Male | 61 | Left MCA | 7.2 |
| 13 | 51 | Female | 72 | Right MCA | 7.2 |
| 14 | 82 | Male | 71 | Right MCA | 8.3 |
| 15 | 79 | Female | 21 | Right MCA | 8.3 |
| 16 | 77 | Female | 68 | Right MCA | 10.4 |
| 17 | 49 | Male | 72 | Left MCA | 8.7 |
| 18 | 79 | Female | 99 | Left MCA | 9.5 |
| 19 | 75 | Male | 63 | Right MCA | 8.3 |
| 20 | 77 | Male | 72 | Right MCA | 7.2 |
| 21 | 84 | Male | 70 | Left MCA | 7.6 |

MCA, middle cerebral artery;

* Wake-up stroke
